# International cooperation. Emerging infectious diseases.

**DOI:** 10.3201/eid0403.980332

**Published:** 1998

**Authors:** J LeDuc

**Affiliations:** Centers for Disease Control and Prevention, Atlanta, Georgia, USA.


### International Cooperation

**James LeDuc**
Centers for Disease Control and Prevention, Atlanta, Georgia, USA

Experts from the World Health Organization (WHO), the European Union (EU), the U.S. Department of Defense (DoD), and other organizations summarized existing and planned collaborations on emerging infectious diseases. The session was chaired by David Heymann, WHO, and James LeDuc, CDC.

One speaker, V. Ramalingaswami, All India Institute of Medical Sciences, India, summarized lessons learned from the plague outbreak in Surat, India. The plague outbreak, the first in many years, found the country ill-prepared to diagnose this disease, and young clinicians lacked experience in recognizing or managing plague-infected patients.

Two speakers examined regional collaborations. Christopher Bartlett, Public Health Laboratory Service, London, summarized the development of international surveillance within EU. The Maastricht Treaty provided the political will to underpin these activities; since the treaty, the heads of European institutes with responsibility for national surveillance have met regularly to assist in strategic development of surveillance activities. Disease-specific networks have been established, each with an operational protocol that sets out agreed case definitions and standard methods and use of information obtained. High quality and timely information is now being provided on a steadily increasing spectrum of infectious diseases through weekly electronic and monthly surveillance bulletin publications. Oyewale Tomori, regional virologist for Africa from WHO, explained that despite substantial advances in disease prevention and control, communicable diseases still constitute a major health problem for Africa. Concern about the deplorable and worsening state of disease control in Africa has led ministers of health in the region to pass several resolutions on prevention of epidemic infectious diseases, yet frequent epidemics continue. Apart from the development of a few disease-specific laboratory diagnostic networks, laboratory services in Africa remain rudimentary and underdeveloped. The WHO Regional Office for Africa has recently formulated a strategic plan for integrated disease surveillance and an action plan to strengthen laboratory capacity in Africa; international support is urgently needed to implement these plans.

Global collaborations were also discussed. Nils Daulaire, U.S. Agency for International Development (USAID), described the recently announced $50 million initiative to address infectious diseases globally. These funds will be used to focus activities on tuberculosis, malaria, antimicrobial resistance, and surveillance, both in countries with USAID missions, as well as regionally. Michael McCarthy, DoD, summarized the new DoD initiative on emerging infectious diseases and explained how the overseas laboratories in Thailand, Kenya, Peru, Brazil, Egypt, and Indonesia will work closely with their host nations and DoD scientists to address emerging endemic disease threats. Maria Neira, WHO, described recent activities to address cholera and other diarrheal diseases of epidemic potential globally. These activities included the secondment of a CDC medical epidemiologist to the WHO subregional office for southern Africa, where in the past several years a plan to improve recognition and response to epidemic diarrheal diseases has been developed and implemented. Included in the program are training on improved patient management, strengthened laboratory capacity, and better communication both within and between countries of southern Africa.

